# Electroacupuncture for slow flow/no-reflow in patients with acute myocardial infarction undergoing percutaneous coronary intervention: a pilot randomized controlled trial

**DOI:** 10.3389/fcvm.2026.1756414

**Published:** 2026-03-10

**Authors:** Xuqiang Wei, Yanbin Peng, Ke Wang, Thomas Krieg, Shiyan Yan, Feng Wu, Min Fan, Jia Zhou

**Affiliations:** 1 Acupuncture Anaesthesia Clinical Research Institute, Yueyang Hospital of Integrated Traditional Chinese and Western Medicine, Shanghai University of Traditional Chinese Medicine, Shanghai, China; 2 Shanghai Acupuncture Clinical Research Center, Shanghai, China; 3 Department of Medicine, Addenbrookes Hospital, University of Cambridge, Cambridge, United Kingdom; 4 International Acupuncture and Moxibustion Innovation Institute, School of Acupuncture-Moxibustion and Tuina, Beijing University of Chinese Medicine, Beijing, China; 5 Department of Cardiovascular Medicine, Yueyang Hospital of Integrated Traditional Chinese and Western Medicine, Shanghai University of Traditional Chinese Medicine, Shanghai, China

**Keywords:** acute myocardial infarction, electroacupuncture, pilot study, randomized controlled trial (RCT), slow flow/no-reflow

## Abstract

**Background:**

Slow flow/no-reflow (SF-NR) complicates up to 44% of percutaneous coronary interventions (PCI) for acute myocardial infarction (AMI), worsening prognosis. Electroacupuncture (EA) may mitigate SF-NR, but clinical evidence is limited.

**Objective:**

This trial was designed to assess the feasibility and effectiveness of intraoperative EA in reducing SF-NR during PCI for AMI patients.

**Design, setting, and participants and interventions:**

This single-center, randomized, assessor-blinded pilot trial enrolled 60 eligible AMI patients undergoing PCI at Yueyang Hospital, China, from August 2023 to March 2024. Participants were randomized to receive PCI with electroacupuncture (EA) stimulating Neiguan (PC6) and Ximen (PC4) acupoints, or PCI alone (control group).

**Main outcomes and measures:**

The primary outcome was the incidence of SF-NR. Secondary outcomes included chest pain (Numerical Rating Scale, NRS), anxiety (Visual Analog Scale for Anxiety, VAS-A), and the occurrence of major adverse cardiac and cerebrovascular events (MACCE) within 30 days, cardiac biomarkers, inflammatory markers.

**Results:**

All 60 patients completed the trial (mean [SD] age, 63.2 [11.4] years; 86.7% male [52/60]). EA significantly reduced SF-NR incidence compared with control (6.7% [2/30] vs. 26.7% [8/30]; RR, 0.2; 95% CI, 0.0 to 0.4; *P* = .04). EA also significantly reduced median pain scores (0 h post-PCI: median difference, −2.5 [95% CI, −3.3 to −0.7]; 12 h post-PCI: median difference, −3.0 [95% CI, −3.5 to −1.9]; both *P* < .001), anxiety scores (0 h post-PCI: median difference, −2.0 [95% CI, −2.8 to −0.2]; 12 h post-PCI: median difference, −2.0 [95% CI, −3.3 to −1.1]; both *P* < .001). No significant differences were found in cardiac biomarkers or 30-day MACCE (16.7% [5/30] vs. 36.7% [11/30]; *P* = .09). However, EA was associated with inflammatory markers at 12 h (Leukocytes, *P* = .03; Neutrophils, *P* = .04; high-sensitivity C-reactive protein, *P* = .03). No adverse events were reported.

**Conclusions:**

Intraoperative EA during PCI was associated with reduced SF-NR and attenuated early inflammation. Improvements in patient-reported pain and anxiety were also observed, though the influence of non-specific effects cannot be ruled out. These preliminary findings demonstrate the feasibility of EA as a PCI adjunct and indicate a potential signal for efficacy, larger multicenter, sham-controlled trials larger multicenter, sham-controlled trials are needed.

**Clinical Trial Registration:**

https://www.chictr.org.cn/, ChiCTR2300072265.

## Highlights

Question: In patients with acute myocardial infarction undergoing percutaneous coronary intervention, does adjunctive intraoperative electroacupuncture, compared with standard care, reduce the incidence of the slow flow/no-reflow phenomenon?
Findings: In this pilot randomized clinical trial that included 60 patients, adjunctive intraoperative electroacupuncture was associated with a lower incidence of the slow flow/no-reflow phenomenon compared with standard percutaneous coronary intervention alone. Patients in the electroacupuncture group also had lower early postoperative levels of some inflammatory markers and reported lower scores for anxiety and pain in this unblinded assessment. These preliminary findings require confirmation in larger, sham-controlled trials.
Meaning: These preliminary findings suggest that intraoperative electroacupuncture may be a feasible and beneficial adjunctive therapy for preventing the slow flow/no-reflow phenomenon and managing perioperative pain and anxiety; larger randomized clinical trials are warranted to confirm these results. The study primarily provides feasibility data and a preliminary efficacy signal to inform the design of such a definitive trial.

## 1 Introduction

Acute myocardial infarction (AMI), caused by acute coronary occlusion due to atherosclerotic plaque rupture or thrombosis, requires urgent revascularization to mitigate myocardial necrosis and restore perfusion (1). Percutaneous coronary intervention (PCI) is the established gold standard for managing AMI, providing rapid reperfusion with minimal invasiveness (2). Nevertheless, up to 44% of patients experience slow flow/no-reflow (SF-NR) following PCI, a condition characterized by compromised coronary microcirculation despite successful epicardial revascularization (3). SF-NR is independently associated with ventricular arrhythmias, heart failure, and increased mortality, significantly reducing the clinical benefits of PCI (4). Research has reported that the SF-NR increases the risk of recurrent infarction fivefold and mortality fourfold, underscoring its critical impact on PCI outcomes (5).

The pathophysiology of SF-NR is multifaceted and involves distal embolization of plaque debris, ischaemia‒reperfusion-induced endothelial dysfunction, inflammatory cytokine cascades, and endothelin-1-mediated vasoconstriction, among other mechanisms (6). Current therapeutic strategies include intracoronary vasodilators, glycoprotein IIb/IIIa inhibitors, and thrombus aspiration (7, 8). However, these interventions often demonstrate inconsistent efficacy, provide only transient benefits, and carry risks of hemorrhagic or thrombotic complications (9, 10). These limitations highlight a pressing unmet need for adjunctive therapies to prevent and manage SF-NR.

Electroacupuncture (EA) has emerged as a promising candidate for addressing this gap, with clinical evidence supporting its potential in cardiovascular care. Perioperative EA has been associated with reduced elevation of myocardial enzymes and improved ventricular remodelling in patients undergoing PCI (11). It has also been shown to be effective in mitigating reperfusion injury and alleviating angina symptoms (12, 13). These findings aligned with its longstanding historical use in acute conditions (14). Mechanistically, preclinical studies further illuminate its biological plausibility. Stimulation of pericardial meridian acupoints [e.g., Neiguan (PC6)] promotes coronary microvascular dilation via activation of the PI3K/Akt/eNOS pathway, increasing nitric oxide bioavailability. Neiguan (PC6) promotes coronary microvascular dilation by activating the PI3K/Akt/eNOS signalling pathway, thereby increasing nitric oxide bioavailability (15–17). Additionally, EA suppresses oxidative stress and inhibits the activation of the NLRP3 inflammasome, reducing the release of proinflammatory cytokines such as interleukin-1β (IL-1β) and tumor necrosis factor-α (TNFα) (18). Despite these findings, no randomized controlled trials (RCTs) have systematically evaluated the efficacy of EA in preventing SF-NR after PCI, leaving a critical evidence gap.

Against this backdrop, advancing directly to large-scale RCTs of EA for SF-NR would be premature. Novel interventions, particularly those integrating traditional therapies into modern procedural settings, require preliminary assessment of feasibility, safety, and procedural logistics before committing to resource-intensive trials. Pilot studies play an indispensable role here: they validate intervention delivery (e.g., intraoperative EA timing, acupoint selection), assess patient acceptability, identify potential protocol refinements, and generate preliminary effect estimates to inform sample size calculations for larger trials.

Thus, we conducted a single-center pilot randomized controlled trial to evaluate intraoperative EA at the Neiguan (PC6) and Ximen (PC4) acupoints, which are acupoints with well-documented cardiovascular effects, as an adjunct to standard PCI compared with PCI alone. We hypothesize that EA reduces the incidence of SF-NR by improving microvascular function through its anti-inflammatory, vasodilatory, and endothelial-protective effects. The secondary objectives included assessing postoperative inflammatory biomarkers, patient-reported chest pain, anxiety, and 30-day major adverse cardiovascular and cerebrovascular events (MACCEs). Using standardized EA parameters (20 Hz, 1 mA) and rigorous outcome assessor blinding, this study aimed to generate critical feasibility data and preliminary evidence to support future multicenter trials.

## 2 Materials and methods

### 2.1 Study design

This was a single-center, parallel-group, randomized controlled pilot trial conducted at the emergency department of Yueyang Hospital, Shanghai University of Traditional Chinese Medicine, from August 1, 2023, to March 1, 2024. The trial enrolled 60 patients with acute myocardial infarction (AMI) who were undergoing percutaneous coronary intervention (PCI). The protocol was aligned with Chinese guidelines for AMI management and approved by the Institutional Ethics Committee of Yueyang Hospital (Approval No. 2023–090-01), adhering to the Declaration of Helsinki. It was prospectively registered at the Chinese Clinical Trial Registry (ChiCTR2300072265), with full protocol details published previously in Supplementary Material 1*.* All participants provided written informed consent prior to enrollment. The study followed the Standards for Reporting Interventions in Clinical Trials of Acupuncture (STRICTA) guidelines (19) and the Consolidated Standards of Reporting Trials (CONSORT) reporting guideline extension for randomized pilot and feasibility trials (20).

### 2.2 Participants

Participants were screened and recruited from the Emergency Department. The eligibility criteria for participants were as follows: (1) aged 18–80 years, regardless of sex; (2) diagnosed with AMI according to the Fourth Universal Definition of Myocardial Infarction (2018) (21) and scheduled for PCI; and (3) provided informed consent. The exclusion criteria were as follows: (1) severe hepatic, renal, or hematopoietic dysfunction (e.g., serum alanine aminotransferase >3× upper normal limit, serum creatinine ≥265 μmol/L); (2) inability to cooperate with or complete the PCI procedure due to alcoholism or cognitive dysfunction; (3) severe cardiac complications (e.g., cardiogenic shock); (4) skin lesions or infections at potential acupuncture sites (precluding safe needle insertion); (5) recent surgery (<4 weeks prior) with bleeding risk; and (6) concurrent participation in other clinical trials. Baseline assessments included 12-lead electrocardiography, cardiac biomarker testing, and routine hematology/biochemistry analyses.

### 2.3 Randomization, allocation concealment, and blinding

An independent statistician, who was not involved in any subsequent participant recruitment, intervention delivery, or outcome assessment, generated the allocation sequence. Using SPSS software (version 25.0; IBM Corp.), a computer-generated 1:1 randomization sequence (block sizes of 4 and 6, randomly varied) was produced. This sequence was then transferred by the statistician onto individual allocation cards. Each card was placed inside a sequentially numbered, opaque, sealed envelope (SNOSE) to ensure allocation concealment. The sealed envelope series was kept under secure custody by a trial coordinator not involved in recruitment. Upon confirming a patient's eligibility and obtaining written informed consent, the research coordinator would retrieve the next sequentially numbered envelope. This envelope was opened by the acupuncturist in a separate room, only after the patient was prepared for the PCI procedure and immediately before the initiation of any study-related intervention (i.e., needle insertion for the EA group). This process ensured that the treatment assignment was concealed until the last possible moment to prevent selection bias.

The blinding of the acupuncturists was not feasible due to the nature of the intervention. To minimize bias, acupuncturists received standardized protocol training, were prohibited from disclosing group assignments or outcomes to participants/study staff, and delivered interventions in physically separate areas to prevent cross-group interaction. The outcome assessors and data analysts remained blinded throughout the trial. Allocation details were restricted to the acupuncturists and the randomization team.

### 2.4 Standard PCI and perioperative care

All patients received guideline-directed medical therapy after PCI. For long-term antiplatelet management post-discharge, the choice between aspirin and clopidogrel monotherapy was per physician discretion and institutional protocols, aligning with contemporary evidence that informs the balance of ischemic and bleeding risks in the maintenance phase (22). PCI was performed under local anaesthesia by ≥2 interventional cardiologists, following the 2021 ACC/AHA/SCAI Coronary Artery Revascularization Guidelines (23). The right radial artery approach was preferred, with femoral access used only for contraindications. Intravenous heparin was administered to maintain an activated clotting time (ACT) > 250 s during the procedure. Stent selection and deployment were determined by angiographic findings and operator discretion. Coronary perfusion was assessed via poststent thrombolysis in myocardial infarction (TIMI) flow grade and corrected TIMI frame count (CTFC) to identify slow flow/no-reflow (SF-NR).

Postprocedural management was conducted in accordance with guideline-directed medical therapy (24), including dual antiplatelet therapy (aspirin 100 mg daily; clopidogrel 75 mg daily), statins, β-blockers, and angiotensin-converting enzyme inhibitors. Radial artery hemostasis was achieved via compression devices, with patients maintained in a supine position. Gradual decompression of the device commenced 2–4 h following PCI, with continuous monitoring of vital signs, including electrocardiography, blood pressure, pulse, oxygen saturation, and assessments for puncture-site bleeding or limb ischemia.

### 2.5 Electroacupuncture intervention

Patients in the EA group received adjunctive EA during PCI, which was administered by licenced acupuncturists with ≥2 years of clinical experience who were trained specifically in the study protocol. Bilateral PC6 and PC4 acupoints, as defined by the Chinese National Standard (GB/T 12346-2006) (Supplementary Figure S1). PC6 is known to modulate cardiovascular function through autonomic regulation and the activation of endothelial nitric oxide synthase activation (25). PC4 was confirmed to stabilize hemodynamics during acute ischemic conditions via stimulation of the vagal nerve (26). The combination of these two acupoints has the potential to enhance cardiovascular function and induce anti-inflammatory, antioxidant, analgesic, and anxiolytic effects (27, 28).

Prior to the procedure, the skin was disinfected, and sterile disposable needles (40 mm × 0.30 mm) were inserted perpendicularly to a depth of 12–20 mm at the left PC6 and PC4. Manual stimulation (lifting-twisting technique) elicited the De Qi sensation (localized soreness or radiating paresthesia). A G6805-2A electroacupuncture device (Shanghai Huayi Co.) delivered continuous 20-Hz pulses at patient-tolerant intensities throughout PCI. The selection of a continuous 20-Hz frequency was based on its established profile in cardiovascular acupuncture research. Preclinical studies have consistently shown that 20-Hz EA stimulation at the Neiguan (PC6) acupoint effectively improves coronary blood flow, reduces infarct size, and attenuates oxidative stress and inflammation in models of myocardial ischemia-reperfusion injury (29, 30). This parameter was chosen to optimize the potential for eliciting the hypothesized cardioprotective and microvascular effects within the acute PCI setting.

### 2.6 Outcome measures

#### 2.6.1 Primary outcome

The primary outcome was the incidence of SF-NR, which was calculated as follows: incidence of SF-NR (%) = (number of cases with no-reflow + number of cases with slow flow)/total number of cases×100%.

SF-NR was defined via the thrombolysis in myocardial infarction (TIMI) flow grade and corrected TIMI frame count (CTFC) criteria and was assessed by two independent interventional cardiologists blinded to group allocation. Slow flow was defined as TIMI grade 2 or CTFC > 27 frames; no-reflow flow was defined as TIMI grade 0–1 or CTFC > 40 frames, excluding vessels with dissection, thrombus, or vasospasm (31). Angiograms were analysed at 30 frames per second, with the CTFC for the left anterior descending artery adjusted by a factor of 1.7 (32). Discrepancies were resolved through consensus.

#### 2.6.2 Secondary outcomes

The secondary outcome was the change from baseline, which included the following: (1) Chest pain was assessed via the numerical rating scale (NRS), where 0 = no pain and 10 = worst imaginable pain. Measurements were taken immediately post-PCI and at 12 h post-PCI. (2) Anxiety: Anxiety was evaluated via the Visual Analogue Scale for Anxiety (VAS-A), with scores ranging from 0 (no anxiety) to 10 (extreme anxiety). Assessments were performed immediately post-PCI and at 12 h after the completion of the PCI procedure. (3) Inflammatory markers: Leukocytes, neutrophils, lymphocytes, and high-sensitivity C-reactive protein (hs-CRP) were measured at 12 and 72 h after the completion of the PCI procedure. (4) Cardiac troponin I (cTnI), myoglobin (Myo), creatine kinase-MB (CK-MB), and N-terminal pro–B-type natriuretic peptide (NT-proBNP) were assessed at 12 and 72 h post-PCI. (5): 30-day major adverse cardiovascular and cerebrovascular events (MACCEs): Comprising hemorrhage, recurrent myocardial infarction, unplanned revascularization, hospital readmission, and cardiogenic death.

While the study protocol initially listed detailed electrocardiogram (ECG) parameters (e.g., ST-segment resolution, QT dispersion) as exploratory secondary outcomes, these were not pursued in the final analysis. This decision, made prior to data analysis, was based on the pilot study's focus on feasibility and core mechanistic signals, coupled with practical challenges in obtaining standardized, high-quality ECG data across sites that would be interpretable in this small sample. The analyzed outcomes listed above were deemed most relevant for informing the design of a subsequent pivotal trial.

### 2.7 Safety monitoring

Acupuncture-related adverse events (AEs), including needle breakage, syncope, puncture-site bleeding, bruising, infection, or localized discomfort, were systematically recorded. Reasons for participant withdrawal were documented throughout the study.

### 2.8 Statistical analysis

Given the exploratory nature of this pilot study and the absence of prior randomized controlled trial data on EA for preventing SF-NR, a formal power calculation for hypothesis testing was not performed. The sample size of 60 participants (30 per group) was determined based on the dual considerations of feasibility and preliminary effect size estimation. A sample of 25–30 participants per group is widely recommended in the methodological literature for pilot trials to adequately assess feasibility parameters such as recruitment rates, protocol adherence, and safety (33). We targeted 30 participants per group, allowing for a 20% over-recruitment buffer to account for potential attrition (34), resulting in a total of 60 participants. This size was deemed sufficient to evaluate the logistical integration of intraoperative EA and to generate reliable estimates of recruitment and retention rates for planning a future definitive trial.

The data analysis was performed by an independent researcher blinded to group allocation. All analyses were conducted with SPSS statistical software (version 25.0, IBM, Inc.). The intention-to-treat principle was applied for the primary outcome. Missing data were imputed via the last observation carried forward method. Continuous variables were assessed for normality via the Shapiro‒Wilk test. Normally distributed data are presented as the means (SDs) and were compared between groups via independent samples t tests and within groups via paired t tests. Nonnormally distributed data are presented as medians [interquartile ranges (IQRs)] and were compared between groups via the Mann‒Whitney U test and within groups via the Wilcoxon signed-rank test. Categorical variables are reported as frequencies and percentages [n (%)] and were compared via the *χ*^2^ test or Fisher's exact test, as appropriate.

The effects of electroacupuncture on the incidence of slow flow/no-reflow and 30-day major adverse cardiovascular and cerebrovascular events (MACCEs) were evaluated via risk ratios (RRs) with 95% CIs. Differences in continuous outcomes between groups from baseline to follow-up were assessed via differences in means or medians with corresponding 95% CIs. All the statistical tests were 2-sided, and a *P* value less than 0.05 was considered statistically significant.

## 3 Results

### 3.1 Baseline characteristics

**Figure 1:**
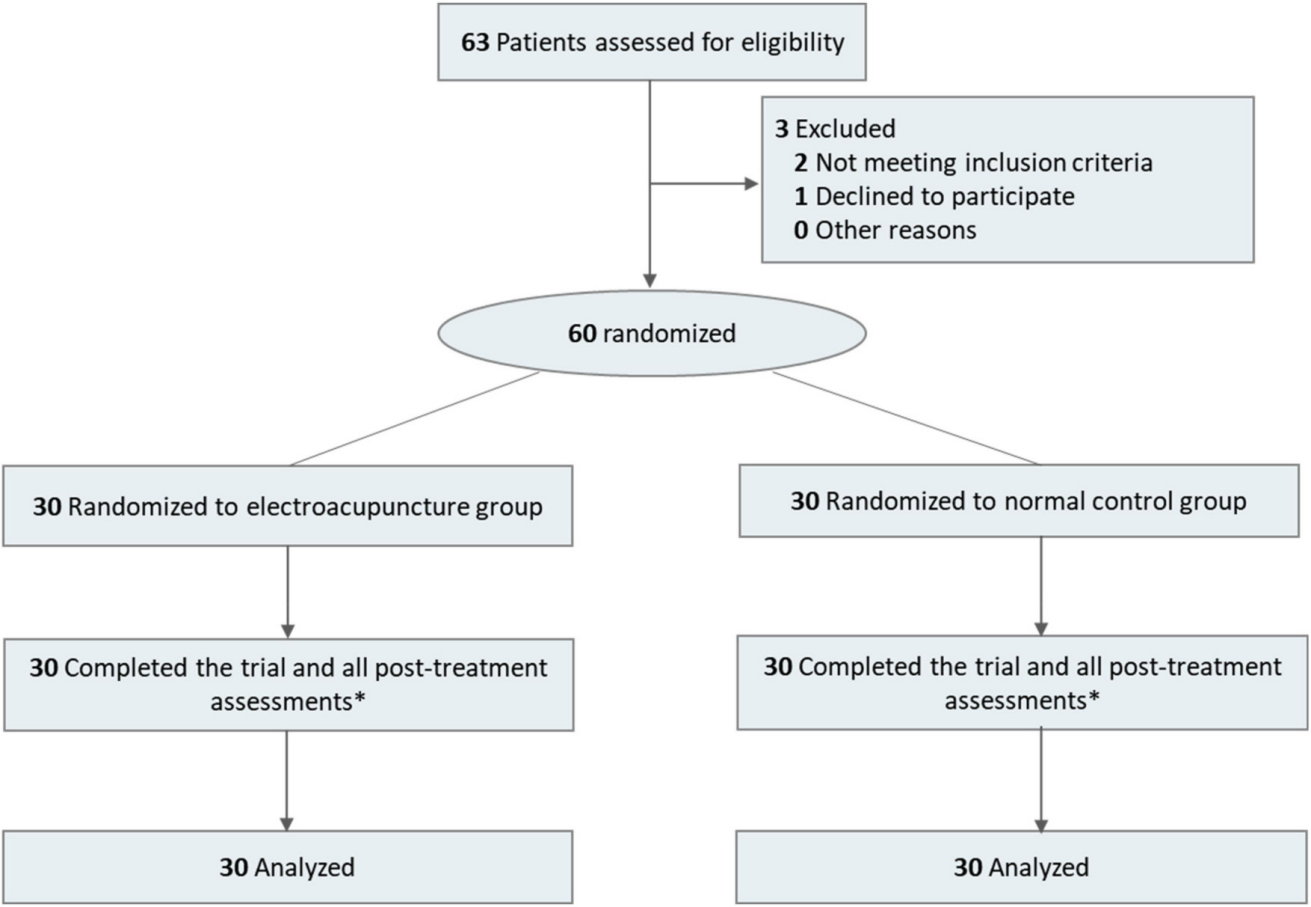
CONSORT flow diagram. *Post-treatment assessments included: incidence of slow flow/no-reflow (primary outcome); Numerical Rating Scale (NRS) for chest pain and Visual Analogue Scale for Anxiety (VAS-A) at 0 h and 12 h post-PCI; inflammatory and cardiac biomarkers at 12 h and 72 h; TCM Symptom Score at 72 h; and 30-day major adverse cardiovascular and cerebrovascular events (MACCEs).

We screened 63 patients with acute myocardial infarction who were receiving percutaneous coronary intervention (PCI). Of these, 60 met the eligibility criteria and were randomized equally to electroacupuncture (EA) or control groups (30 per group; Figure 1). The participants had a mean [SD] age of 63.2 [11.4] years, with 52 male participants (86.7%) and 8 female participants (13.3%). No dropouts or intervention-related adverse events occurred. Baseline demographic and clinical characteristics were well balanced between the groups (Table 1). It is noted that the preoperative pain score (NRS) showed a difference of borderline statistical significance (*P* = 0.05), with a higher median score in the EA group.

**Table 1:** Baseline characteristics.

Covariates	Patients (*N* = 60)	Electroacupuncture group (*n* = 30)	Control group (*n* = 30)	*P* value
Age, mean (SD), year	63.2 (11.4)	62.7 (12.3)	63.7 (10.6)	.74
Gender (*n*, %)				
Male	52 (86.7)	26 (86.7)	26 (86.7)	.39
Female	8 (13.3)	4 (13.3)	4 (13.3)	
BMI, mean (SD)	25.1 (3.7)	24.3 (2.8)	25.8 (4.3)	.13
Smoking history (*n*, %)	35 (58.3)	17 (56.7)	18 (60.0)	.79
Alcohol history (*n*, %)	13 (21.7)	8 (26.7)	5 (16.7)	.35
Exercise ≥30 min, 3 times/w (*n*, %)	12 (20.0)	7 (23.3)	7 (23.3)	1.00
Time from Onset to PCI (h), median (IQR)	5 (2 to 40)	11.5 (2 to 48)	4 (2–30)	.29
Comorbidities (*n*, %)				
Hypertension	31 (51.7)	19 (63.3)	12 (40.0)	.07
Hyperlipidemia	8 (13.3)	5 (16.7)	3 (10.0)	.45
Diabetes	18 (30.0)	8 (26.7)	10 (33.3)	.57
Coronary heart disease	7 (11.7)	3 (10.0)	4 (13.3)	.69
Laboratory tests				
ALT (U/L), median (IQR)	33.0 (22.3 to 47.8)	36.5 (24.8 to74.9)	30.0 (20.8 to 57.5)	.17
AST(U/L), median (IQR)	38.5 (29.0 to 128.5)	35.5 (28.8 to 164.5)	40.0 (28.5 to 282.7)	.90
Urea (mmol/L), median (IQR)	5.5 (4.7 to 7.3)	5.3 (4.8 to 7.4)	5.6 (4.2 to 6.6)	.52
Creatinine (umol/L), median (IQR)	70.0 (59.8 to 84.1)	70.4 (60.7 to 88.8)	70.0 (57.1 to 80.7)	.88
Uric Acid (umol/L), mean (SD)	357.4 (112.7)	377.3 (136.1)	341.8 (78.6)	.12
Blood Potassium (umol/L),mean (SD)	3.9 (0.5)	3.9 (0.5)	4.0 (0.5)	.47
Leukocytes (10^9/L), median (IQR)	10.0 (8.3 t0 12.0)	10.0 (8.7 to 13.9)	10.0 (7.9 to 11.5)	.40
Erythrocyte (10^9/L), mean (SD)	4.9 (0.6)	4.9 (0.6)	4.9 (0.5)	.90
Neutrophils (10^9/L), median (IQR)	6.7 (5.2 to 8.6)	6.8 (4.9 to 9.8)	6.3 (5.4 to 8.1)	.80
Lymphocytes (10^9/L), median (IQR)	2.1 (1.4 to 2.8)	2.1 (1.3 to 2.7)	2.2 (1.5 to 2.8)	.66
Monocytes (10^9/L), mean (SD)	0.76 (0.30)	0.81 (0.73)	0.71 (0.27)	.63
Thrombocyte, mean (SD)	242.9 (65.9)	242.7 (73.1)	243.1 (59.0)	.98
hs-CRP (mg/L), median (IQR)	3.7 (1.6 to 11.0)	5.3 (2.1 to 18.0)	2.9 (1.2 to 7.4)	.18
cTnI (ng/mL) median (IQR)	1.1 (0.2 to 2.7)	1.7 (0.5 to 6.8)	0.4 (0.2 to 2.3)	.06
Myoglobin (ng/mL), median (IQR)	49.1 (15.0 to 106.6)	52.3 (15.0 to 142.7)	46.9 (15.0 to 91.7)	.95
NT-proBNP (pg/mL), median (IQR)	127.6 (58.2 to 635.5)	139.5 (78.9 to 83.5)	127.6 (25.0 to 456.8)	.29
CK-MB (ng/mL), median (IQR)	7.9 (1.6 to 18.8)	8.7 (1.3 to 28.1)	6.4 (2.3 to 15.1)	.38
NRS (score), median (IQR)	8 (6 to 10)	9 (7 to 10)	6 (6 to 9)	.05
VAS-A (score), mean (SD)	4.8 (2.43)	5.2 (2.2)	4.4 (2.6)	.20
Preoperative TIMI grade (*n*, %)				
0	32 (53.3)	17 (56.7)	15 (50.0)	.95
1	21 (35.0)	10 (33.3)	11 (36.7)	
2	5 (8.3)	2 (6.7)	3 (10.0)	
3	2 (3.3)	1 (3.3)	1 (3.3)	
CTFC, mean (SD)	63.8 (14.8)	64.2 (15.2)	63.5 (14.6)	.87
Culprit Vessel (*n*, %)				
Left Anterior Descending	30 (50.0)	12 (40.0)	18 (60.0)	.18
Left Circumflex	8 (13.3)	6 (20.0)	2 (6.7)	
Right Coronary	22 (36.7)	12 (40.00)	10 (33.3)	
Number of Diseased Vessels (*n*, %)				
Single Vessel	51 (85.0)	24 (80.0)	27 (90.0)	.43
Double Vessel	8 (13.3)	5 (16.7)	3 (10.0)	
Triple Vessel	1 (1.7)	1 (3.3)	0 (0.0)	
Medication				
Antiplatelet and Anticoagulant Drugs (*n*, %)	7 (11.7)	3 (10.0)	4 (13.3)	.69
Statin Drugs	3 (5.0)	1 (3.3)	2 (6. 7)	.55
ACEI/ARB	8 (13.3)	5 (16.7)	3 (10.0)	.45
CCB	5 (8.33)	2 (6.7)	3 (10.0)	.64
Beta-Blockers	1 (1.7)	1 (3.3)	0 (0.0)	.31
Nitrate Drugs	9 (15.0)	4 (13.3)	5 (16.7)	.72
Diuretics	2 (3.3)	1 (3.3)	1 (3.3)	.79
AMI type (*n*, %)				
ST	31 (51.7)	17 (56.7)	14 (46.7)	.44
NST	29 (48.3)	13 (43.3)	16 (53.3)	
Number of Stents Implanted (*n*, %)				
None	18 (30.0)	9 (15.0)	9 (15.0)	.46
One	35 (58.3)	16 (26.6)	19 (31.7)	
Two	7 (11.7)	5 (8.3)	2 (3.3)	
Thrombus Aspiration (*n*, %)	25 (41.7)	13 (43.3)	12 (40.0)	.79
GP IIb/IIIa Receptor Antagonists (*n*, %)	33 (55.0)	16 (53.3)	17 (56.7)	.79

NRS, Numerical Rating Score; VAS-A, Visual Analogue Scale for Anxiety; TIMI, Thrombolysis In Myocardial Infarction; CTFC, Corrected TIMI frame count; CCB, Calcium-channel Blockers; STEMI, ST-elevation myocardial infarction; NSTEMI, non-ST-elevation myocardial infarction.

### 3.2 Primary outcome

The EA group demonstrated a significantly lower intraoperative slow-flow/no-reflow (SF-NR) incidence: 2 patients (6.7%; 1 slow-flow, 1 no-reflow) vs. 8 patients (26.7%; 6 slow-flow, 2 no-reflow) in the control group. This corresponded to a 20% relative risk reduction [risk ratio (RR) 0.2; 95% CI, 0.0–0.4; *P* = 0.04] (Table 2; Supplementary Table S1).

**Table 2:** Primary outcome and patient-reported outcomes.

Outcome	Electroacupuncture group (*n* = 30)	Control group (*n* = 30)	Between-group difference (95%CI)	*P* value
Primary outcome				
SF-NR incidence (*n*, %)	2 (6.7)	8 (26.7)	0.2 (0.0 to −0.4)^a^	.04
Secondary outcomes				
NRS, median (IQR), 0 h post-PCI	3.5 (2.0 to 4.0)	6.0 (4.0 to 6.0)	−2.5 (−3.3 to −0.7)^b^	<.001
VAS-A, median (IQR), 0 h post-PCI	2.0 (1.0 to 3.3)	4.0 (3.0 to 6.0)	−2.0 (−2.8 to −0.2)^b^	.004
NRS, median (IQR), 12 h post-PCI	1.0 (0.0 to 2.0)	4.0 (3.0 to 5.00)	−3.0 (−3.5 to −1.9)^b^	<.001
VAS-A, median (IQR), 12 h post-PCI	1.0 (0.8 to 2.0)	3.0 (2.0 to 5.0)	−2.0 (−3.3 to −1.1)^b^	<.001
TCM Scores, median (IQR), 72 h post-PCI	3.5 (2.0 to 5.0)	5.0 (4.0 to 7.0)	−1.5 (−0.5 to 3.5)	0.157

CI, confidence interval; SD, standard deviation; SF-NR, slow flow/no-reflow; VAS-A, visual analogue scale for anxiety.

^a^ Risk ratio (95% CI);

^b^ Difference in medians.

### 3.3 Secondary outcomes

Compared with baseline, electroacupuncture significantly reduced chest pain (NRS) and anxiety (VAS-A) at both 0 h post-PCI and 12 h post-PCI. At 0 h post-PCI, the median NRS score was 3.50 (IQR, 2.0–4.0) in the electroacupuncture group and 6.00 (IQR, 4.0–6.0) in the control group [mean difference (MD), −2.50; 95% CI, −3.3 to −0.7; *P* < 0.001]. At 12 h post-PCI, the NRS score in the EA group further improved (MD, −3.00; 95% CI, −3.5 to −1.9; *P* < 0.001).

**Figure 2:**
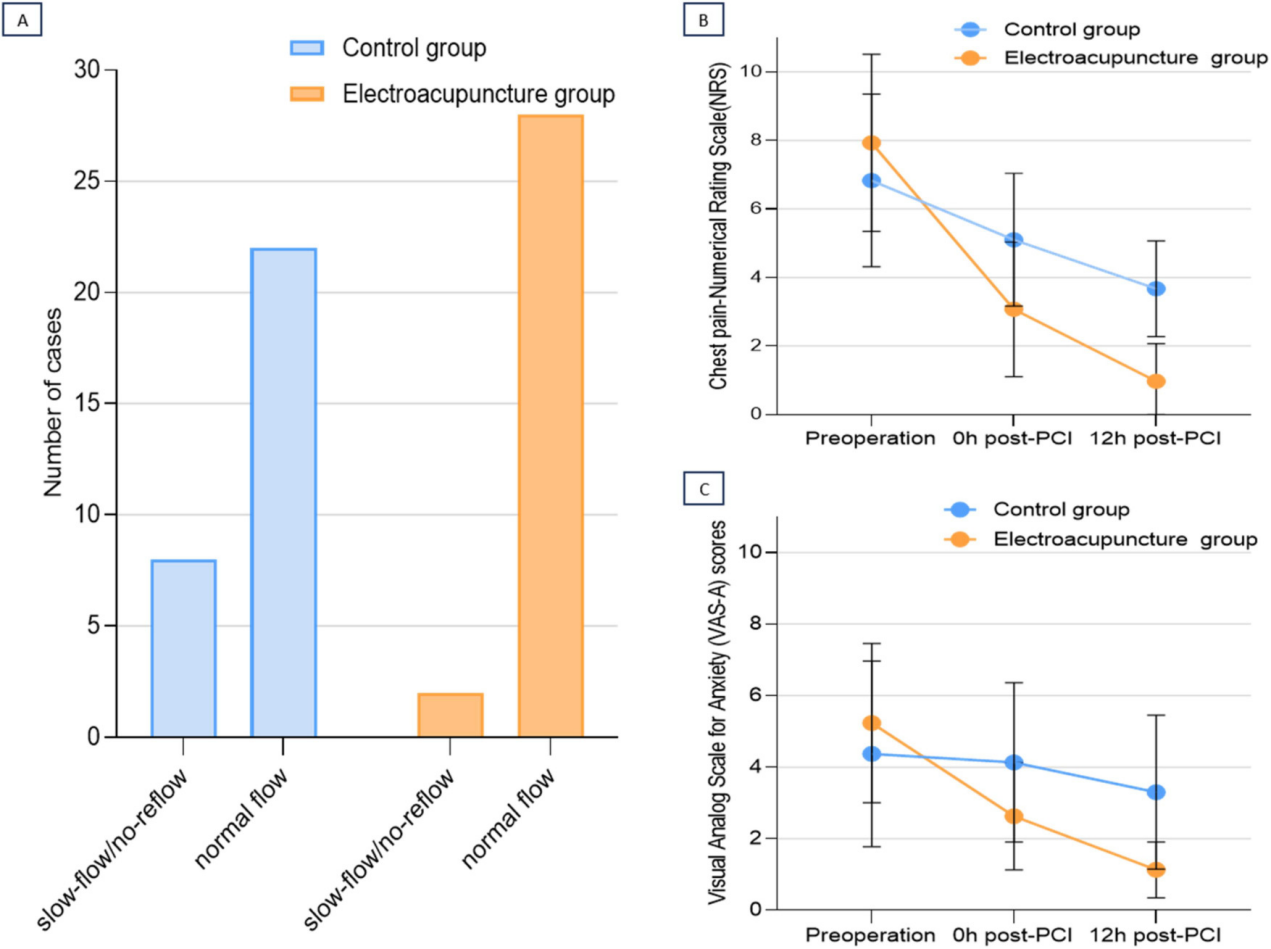
Primary and patient-reported outcomes. **(A)** Bar graph showing the number of patients experiencing slow flow/no-reflow (SF-NR) in the Electroacupuncture (EA) and Control groups. **(B)** Line graph depicting chest pain intensity scores (Numerical Rating Scale, NRS; range 0–10) assessed at preoperative baseline, immediately post-PCI (0 h), and 12 h post-PCI. **(C)** Line graph depicting anxiety scores (Visual Analogue Scale for Anxiety, VAS-A; range 0–10) assessed at the same three time points. In panels B and C, data points represent the mean score, and error bars represent the standard error of the mean (SEM), details of statistical tests and exact *P* values are provided in the main text and Table 2.

VAS-A scores demonstrated similar reductions in the EA group compared with the control group: at 0 h post-PCI, the median score was 2.00 (IQR, 1.0–3.3) vs. 4.0 (IQR, 3.0–6.0), and the difference between the two groups was −2.00 (95% CI, −2.8–0.2; *P* = 0.004). At 12 h post-PCI, the EA group showed greater improvement in the MD (2.0; 95% CI, −3.3 to 1.1; *P* < 0.001) (Table 2; Figure 2).

**Figure 3:**
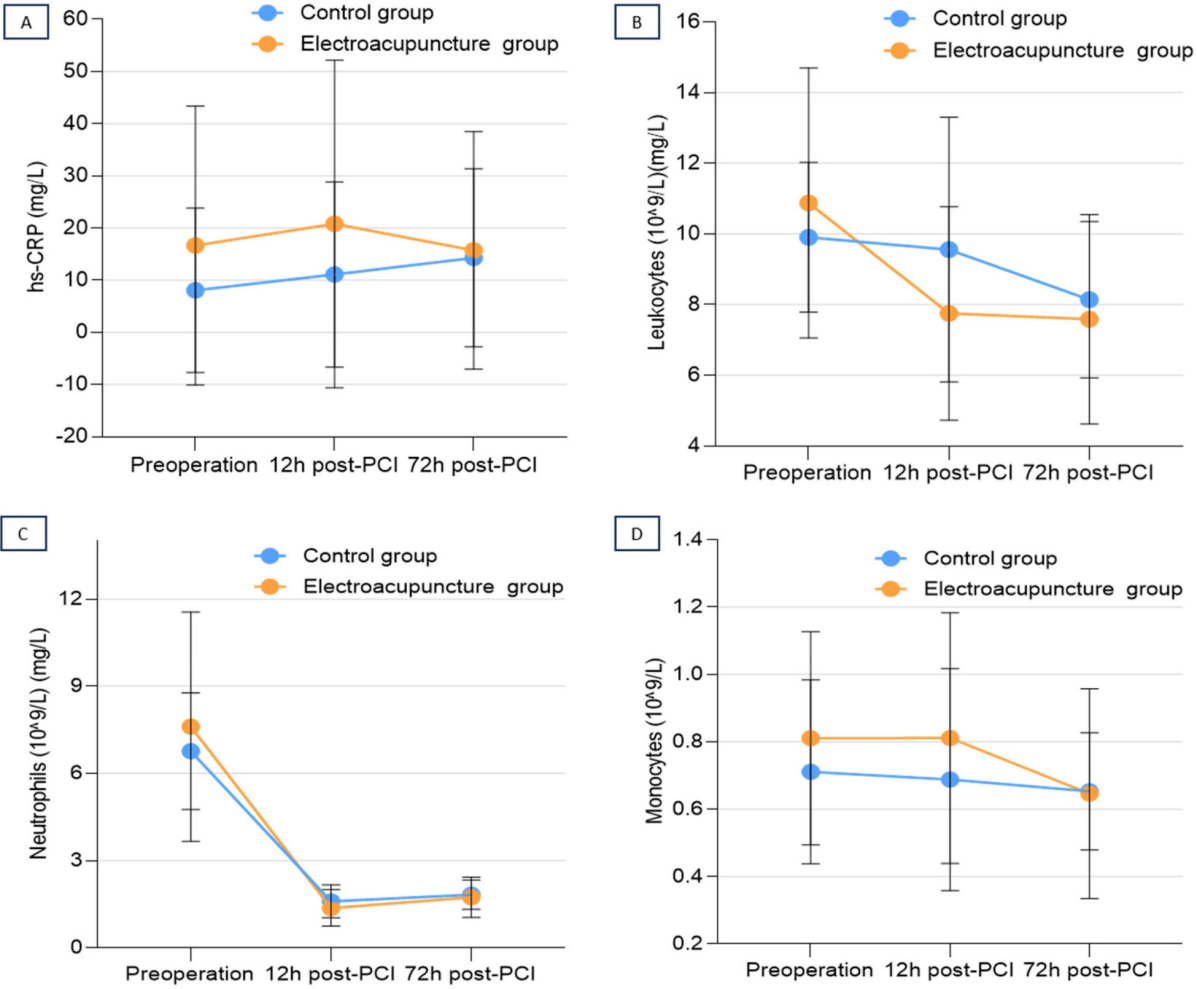
Perioperative inflammatory biomarker profiles. Line graphs showing the perioperative time course of **(A)** high-sensitivity C-reactive protein (hs-CRP), **(B)** leukocyte count, **(C)** neutrophil count, and **(D)** monocyte count in the lectroacupuncture (EA) and Control groups. Blood samples were taken at preoperative baseline, 12 h post-PCI, and 72 h post-PCI. Data points represent the mean concentration or count, and error bars represent the standard error of the mean (SEM). Note the different *Y*-axis scales for each biomarker. Significant between-group differences at specific time points (e.g., 12 h for hs-CRP, leukocytes, and neutrophils) are reported with exact *P* values in the main text and Table 3.

Inflammatory biomarkers at 12 h post-PCI showed significant reductions in the EA group compared to control: hs-CRP (median, 3.9 vs. 7.3 mg/L; *P* = .03), leukocytes (median, 7.5 vs. 9.0 × 10⁹/L; *P* = .03), and neutrophils (mean/median, 5.4 vs. 6.5 × 10⁹/L; *P* = .04). However, these between-group differences were no longer present at 72 h post-PCI (all *P* > 0.05) (Table 2; Figure 3). Additionally, there was no significant between-group difference in the TCM Symptom Scores at 72 h post-PCI (EA group: median 3.5 [IQR, (2.0 to 5.0)] vs. Control group: median, 5.0 [IQR (4.0 to 7.0)], *P* = 0.157).

**Table 3:** Laboratory biomarkers and clinical events.

Outcome	Electroacupuncture group (*n* = 30)	Control group (*n* = 30)	Between-group difference (95%CI)	*P* value
Cardiac biomarkers, 12 h post-PCI				
cTnI, median (IQR)	1.8 (0.5 to 6.1)	1.9 (0.8 to 4.3)	0.8 (−2.3 to 3. 8)^b^	.83
Myo, median (IQR)	10.0 (10.0 to 80.4)	10.0 (10.0 to 105.6)	−21.3 (−113.9 to 156.4)^b^	.56
CK-MB, median (IQR)	38.5 (7.9 to 95.7)	30.51 (7.7 to 110.20)	−4.8 (−871.1 to 1,995.8)^b^	.59
NT-proBNP, median (IQR)	683.0 (248.0 to 1487.5)	543.8 (191.5 to 1102.5)	−562.4 (−871.1 to 1,995.8)^b^	.89
Inflammatory biomarkers, 12 h post-PCI				
hs-CRP, median (IQR), mg/L	3.9 (1.4 to 14.2)	7.3 (3.2 to 21.3)	−9.7 (−23.0 to −3.6)^b^	.03
Leukocytes, median (IQR), 10^9/L	7.5 (6.2 to 9.4)	9.0 (7.1 to 10.6)	−1.8 (−3.6 to −0.1)^b^	.03
Neutrophils, median (IQR), 10^9/L	5.4 (4.0 to 6.7)	6.5 (4.9 to 7.9)	−1.1 (−2.0- to −0.0)^c^	.04
Lymphocytes, median (IQR), 10^9/L	1.2 (1.0 to 1.8)	1.6 (1.2 to 1.9)	0.2 (−0.1 to 0.5)^b^	.08
Cardiac biomarkers, 72 h post-PCI				
cTnI, median (IQR)	0.2 (0.02 to 0.9)	0.6 (0.2 to 1.3)	0.7 (−0.3 to 1.7)^b^	.08
Myo, median (IQR)	10.0 (10.0 to 31.0)	10.0 (10.0 to 47.3)	13.0 (−37.1 to 63.1)^b^	.82
CK-MB, median (IQR)	2.9 (1.0 to 5.3)	2.8 (1.2 to 7.4)	1.8 (−14.2 to 17.7)^b^	.61
NT-proBNP, median (IQR)	254.0 (106.0 to 762.5)	476.2 (17.5 to 936.1)	350.1 (−88.3 to 788.4)^b^	.12
Inflammatory biomarkers, 72 h post-PCI				
hs-CRP, median (IQR), mg/L	6.3 (2.3 to 16.3)	7.9 (2.7 to 17.2)	−1.4 (−11.8 to 9.0)^b^	.54
Leukocytes, mean (SD), 10^9/L	7.6 (3.0)	8.1 (2.2)	0.6 (−0.8 to 1.9)^c^	.22
Neutrophils, mean (SD), 10^9/L	5.0 (2.4)	5.2 (1.4)	0.3 (−0.8 to 1.3)^c^	.46
Lymphocytes, median (IQR), 10^9/L	1.6 (1.2 to 2.1)	1.8 (1.6 to 2.1)	0.1 (−0.2 to 0.4)^b^	.21
30-Day MACCEs (*n*, %)	5 (16.7)	11 (36.7)	0.3 (0.1 to 1.2)^a^	.09
Adverse events	0	0	NA	NA

CK-MB, creatine kinase-MB; cTnI, cardiac troponin I; hs-CRP, high-sensitivity C-reactive protein; IQR, interquartile range; MACCEs, major adverse cardiovascular and cerebrovascular events; Myo, myoglobin; NRS, numerical rating scale; NT-proBNP, N-terminal pro-B-type natriuretic peptide; PCI, percutaneous coronary intervention; NA, Not Applicable.

^a^ Risk ratio (95% CI);

^b^ Difference in medians;

^c^ Difference in means.

No significant between-group differences were detected in cardiac markers (cTnI, myoglobin, CK-MB, and NT-proBNP) or lymphocyte counts at either timepoint (*P* > 0.05 for all). The 30-day MACCE incidence was 16.7% (5/30) with electroacupuncture vs. 36.7% (11/30) with the control (risk ratio, 0.3; 95% CI, 0.1–1.2; *P* = 0.09). No acupuncture-related adverse events occurred (Table 3).

## 4 Discussion

In this pilot randomized controlled trial (RCT), we reported that compared with standard care alone, intraoperative electroacupuncture (EA) as an adjunct to percutaneous coronary intervention (PCI) was associated with a significantly lower incidence of slow-flow/no-reflow (SF-NR) in patients with acute myocardial infarction (AMI) (6.7% vs. 26.7%; *P* = .04). This corresponds to an absolute risk reduction of 20% and a number needed to treat (NNT) of 5. While this NNT suggests a potentially meaningful clinical effect, it is crucial to emphasize that this is a very preliminary estimate derived from a small pilot trial with a limited number of events. The wide confidence interval around the risk ratio underscores the uncertainty in this estimate, and its reproducibility and true clinical impact must be validated in a larger, definitive trial. Additionally, the EA group demonstrated reductions in inflammatory markers (leukocytes, neutrophils, and hs-CRP), improvements in chest pain and anxiety, and no adverse events. These preliminary findings from a pilot study suggest that EA may warrant further investigation as a safe, nonpharmacologic adjunct to PCI for AMI patients. However, given the small sample size (*N* = 60), these results are hypothesis-generating, and confirmation in larger, sham-controlled studies with longer follow-up is essential.

The observed improvements in post-PCI thrombolysis in myocardial infarction (TIMI) flow grade and corrected TIMI frame count (CTFC) in the EA group support a potential role for EA in enhancing coronary perfusion. This finding aligns with prior evidence that acupuncture modulates neuroendocrine pathways, suppresses inflammation (18), and improves endothelial nitric oxide bioavailability (35). These mechanisms may ameliorate microvascular dysfunction and improve perfusion. Consequently, the incidence of SF-NR could decrease.

EA also significantly reduced post-PCI chest pain (NRS) and anxiety (VAS-A), which is consistent with its established analgesic and anxiolytic properties. Preclinical evidence suggests potential mechanisms, such as endogenous opioid release, hypothalamic‒pituitary‒adrenal (HPA) axis modulation, and vagal nerve activation (36–38). These pathways are clinically significant in AMI, in which pain and anxiety exacerbate haemodynamic instability. Such exacerbation may subsequently hinder patient recovery. The NRS and VAS-A scales were selected for their reliability in quantifying symptom severity (39). Notably, the anxiolytic efficacy of EA surpassed that reported in Armond et al.'s trial (40), potentially owing to differences in assessment tools (disease-specific vs. generic scales), stimulation parameters (e.g., acupoint selection, frequency), or postoperative observation windows. However, the interpretation of these patient-reported improvements must be tempered by the study's design limitations, chiefly the lack of patient blinding and a sham control group, which precludes distinguishing specific treatment effects from potent contextual or placebo responses. Another deatais should also consider the observed, albeit borderline, higher preoperative pain score in this group at baseline. While the statistical adjustment for baseline scores in longitudinal analysis supports a treatment effect, this baseline difference underscores the importance of randomization in larger trials to ensure full baseline comparability for subjective endpoints.

In the context of traditional Chinese medicine (TCM) evaluation, we did not observe a significant difference in TCM symptom scores between groups at 72 h. This may suggest that a single intraoperative EA session, while showing effects on specific physiological parameters (microvascular flow, inflammation) and acute symptoms (pain, anxiety), did not induce a measurable modulation of the broader, integrative symptom complex as defined by TCM theory within the short observation window. It is also possible that the TCM symptom scale used, while validated, may not be sensitive enough to detect changes in this acute, highly managed post-PCI setting, or that the effects on TCM-defined syndromes manifest over a longer period. This finding highlights the complexity of bridging disease-specific physiological outcomes with holistic TCM assessments and suggests that future trials integrating TCM outcomes might consider longer follow-up or different assessment tools.

Consistent with the documented anti-inflammatory effects of EA (41), we observed reductions in leukocyte counts, neutrophil‒lymphocyte ratios, and high-sensitivity C-reactive protein (hs-CRP) levels. Lymphocyte counts did not significantly change, potentially reflecting limited statistical power or pathway-specific modulation. The anti-inflammatory effect observed with EA invites comparison with contemporary pharmacological strategies for post-MI inflammation. Recent evidence on targeted anti-inflammatory therapy provides a relevant context. A 2025 meta-analysis of colchicine, a potent anti-inflammatory agent, concluded that it did not significantly reduce major adverse cardiovascular events or hs-CRP levels in post-MI patients compared to placebo, yet it was associated with a significantly increased risk of gastrointestinal adverse events (42). While direct comparisons are not possible, EA's observed anti-inflammatory effect, coupled with an absence of serious adverse events in our trial, suggests it may represent a safe complementary strategy for managing post-MI inflammation, meriting direct evaluation against pharmacological agents.

We monitored cardiac markers at 12 h (peak levels) and 72 h (prognostic relevance) postadmission, as previously validated^44^. The transient nature of the anti-inflammatory effect—observed at 12 h but absent at 72 h—merits discussion. This pattern is consistent with several plausible explanations: (a) the natural resolution of the acute post-procedural inflammatory surge overrides any early intervention effect; (b) a single intraoperative EA session may exert only a time-limited biological modulation; or (c) the study was underpowered to detect sustained but smaller differences at later time points. Future studies with repeated EA sessions and more frequent biomarker sampling are needed to distinguish between these possibilities.

Notably, despite improvements in perfusion and inflammation, EA did not alter cardiac biomarkers (cTnI, Myo, CK-MB, and NT-proBNP). This finding suggests that EA's primary benefit may lie in enhancing microcirculatory flow rather than directly attenuating myocardial injury, a hypothesis supported by preclinical models showing that acupuncture accelerates postischemic microcirculatory recovery without reducing infarct sizes (43). This contrasts with studies reporting acupuncture-related improvements in cardiac markers (44), highlighting the need for further research into context-specific mechanisms (e.g., timing of intervention, patient characteristics). The observed dissociation between improved microvascular perfusion and unchanged myocardial injury biomarkers warrants careful interpretation. While adjunctive EA significantly reduced the incidence of SF-NR and improved TIMI flow grade, this hemodynamic benefit did not translate into a statistically significant reduction in cardiac Troponin I or CK-MB levels. We hypothesize that the magnitude or duration of the hemodynamic improvement may have been too modest within the context of this pilot study to meaningfully alter the final infarct size. Alternatively, the timing of the single intraoperative EA session may not have sufficiently preceded the completion of the ischemic cascade to salvage myocardium at risk. This finding is consistent with the emerging concept that improving microvascular flow (reducing “no-reflow”) and limiting infarct size, while related, may be distinct therapeutic targets with potentially different underlying mechanisms. Therefore, while EA shows potential for improving coronary microcirculation, our current findings do not demonstrate a clear effect on infarct size reduction. Future studies with larger cohorts, serial cardiac imaging (e.g., cardiac MRI) to quantify myocardial salvage, and potentially adjusted intervention timing are warranted to fully elucidate EA's role in myocardial protection post-PCI.

Slow-flow/no-reflow (SF-NR) is associated with adverse cardiac remodelling and increased incidence of major adverse cardiovascular and cerebrovascular events (MACCEs). Consequently, we assessed the 30-day MACCE incidence to evaluate the effects of sustained treatment. Although the reduction in 30-day MACCE incidence did not reach statistical significance, the observed substantial decrease represents a clinically meaningful trend. This finding is likely attributable to the limited statistical power of the small sample size and short follow-up duration. These findings reinforce the need for larger trials with extended follow-up to evaluate the impact of EA on long-term prognosis.

## 5 Strengths and limitations of the study

### 5.1 Strengths

This study addresses the clinically significant problem of SF-NR in AMI patients undergoing PCI and explores electroacupuncture as a novel adjunctive therapy. The study demonstrates the feasibility of intraoperative electroacupuncture with a 100% completion rate and no adverse events, which is a strong positive for the potential of this intervention in clinical practice.

### 5.2 Limitations

This study has several important limitations that warrant careful consideration.

First, this was a single-center, small-sample pilot randomized trial, designed to assess feasibility, safety, and preliminary signal detection rather than to establish clinical efficacy. The limited sample size resulted in a small number of primary outcome events, yielding imprecise effect estimates with wide confidence intervals. Specifically, the risk ratio for the primary outcome was 0.2 with a 95% confidence interval of 0.0 to 0.4, which includes values close to the null effect. Accordingly, the observed between-group difference in SF-NR incidence should be interpreted with caution and cannot be considered definitive evidence of efficacy. Importantly, the study was underpowered for secondary clinical outcomes, including 30-day MACCEs.

Second, and most critically, blinding limitations constitute the fundamental methodological weakness of this study. Although outcome assessors and data analysts were blinded, blinding of the acupuncturists delivering EA and the patients receiving it was not feasible. Furthermore, the control group did not undergo a comparable adjunctive procedure, creating a stark contrast in patient experience. This design likely introduced substantial performance bias and potent placebo/expectancy effects, particularly for the patient-reported outcomes of pain and anxiety. As discussed in detail above, this means the apparent benefits for NRS and VAS-A may largely reflect non-specific psychological effects rather than a specific treatment effect of EA. Therefore, future definitive trials must incorporate a credible sham acupuncture control (e.g., non-penetrating needles at non-acupoints or superficial needle insertion at non-acupoints) to reliably distinguish the specific physiological effects of EA from the contextual effects of the therapeutic encounter.

Third, although transient between-group differences in selected inflammatory markers were observed at 12 h post-PCI, these differences were not sustained at 72 h. This pattern may reflect the natural resolution of post-procedural inflammation, a short-lived effect of a single intraoperative electroacupuncture session, or insufficient statistical power at later time points. Accordingly, these findings should be interpreted as exploratory and time-limited and do not support conclusions regarding sustained anti-inflammatory effects.

Fourth, despite an observed reduction in angiographic slow flow/no-reflow, this did not translate into measurable reductions in myocardial injury biomarkers, such as cardiac troponin or CK-MB. This dissociation suggests that any improvement in microvascular perfusion was likely modest, transient, or occurred too late to influence infarct size. Therefore, the present study does not provide evidence of myocardial protection. Moreover, we did not analyze comprehensive ECG parameters as initially contemplated in the protocol. While this decision reflected a focused prioritization of resources on outcomes central to the pilot's feasibility and signal-finding goals, it means we cannot comment on EA's potential electrophysiological effects. Future larger-scale trials should incorporate robust, protocol-driven ECG assessments with core laboratory adjudication to explore this aspect.

Finally, the external validity and generalizability of our findings are fundamentally constrained by the study population. Participants were predominantly male (86.7%) and recruited from a single tertiary care center in China. This homogeneity means that the observed feasibility, safety profile, and preliminary effect sizes may not be directly applicable to other important demographic groups, particularly women, or to different healthcare systems and clinical practice settings. Future definitive trials must prioritize multicenter designs with deliberate efforts to enroll more diverse and representative patient populations.

## 6 Conclusions

In this pilot randomized trial, intraprocedural electroacupuncture was feasibly and safely integrated into the PCI workflow. Preliminary results indicated improved post-procedural coronary flow in the electroacupuncture group, although no significant differences were observed in myocardial injury biomarkers or in patient-reported pain and anxiety outcomes. While these findings suggest potential benefits, the small sample size and absence of a sham control require cautious interpretation. This study establishes the logistical integration of EA within the PCI setting and provides the necessary foundation and effect size estimates to inform the design of future, adequately powered, multicenter, sham-controlled randomized trials.

## Data Availability

Due to the nature of the clinical data and patient privacy regulations in China, the complete de-identified individual participant data cannot be made publicly available. However, the data supporting the main findings are available within the article and its supplementary files. Additional aggregated data or analytic code will be shared upon reasonable request to the corresponding authors, subject to review and approval of a proposal and the execution of a data use agreement.
