# Supplementary material for: Electroacupuncture for slow flow/no-reflow in patients with acute myocardial infarction undergoing percutaneous coronary intervention: a pilot randomized controlled trial
Source: Front Cardiovasc Med. 2026 Mar 10;13:1756414. doi: 10.3389/fcvm.2026.1756414 (PMC13014618; doi:10.3389/fcvm.2026.1756414)
Supplement: Supplementary file 2 [file Datasheet2.docx]

**eFigure 1 Location of acupoint**


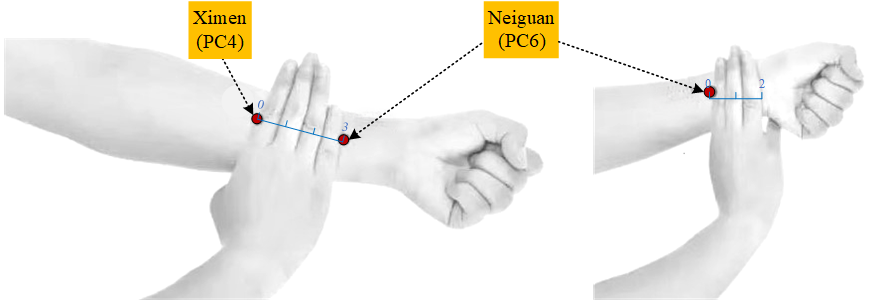


| **eTable 1. Comparison of the incidence of SF-NR between two groups** | | | | |
| --- | --- | --- | --- | --- |
| **Outcome** | **Electroacupuncture group (n=30)** | **Control group (n=30)** | **Between-group difference (95%CI)** | **P value** |
| **TIMI grading afterPCI (n,%)**^a^ | | | | |
| 0 | 0 (0.0) | 1 (3.3) | 0.0 (-0.1 to 0.0) | 0.93 |
| 1 | 1 (3.3) | 1 (3.3) | 0.0 (-0.1 to 0.1) |  |
| 2 | 1 (3.3) | 4 (13.3) | 0.1 (-0.3 to 0.0) |  |
| 3 | 28 (93.3) | 24 (80.0) | 0.7 (0.6 to 0.9) |  |
| **CTFC after PCI, mean (SD)**^b^ | 22.3 ± 4.0 | 25.3 ± 6.2 | -3.0 (-5.7 to -0.3) | .03 |
| **SF-NR incidence**^a^ | 2 (6. 7) | 8 (26.7) | 0.20 (0.0 to 0.4) | .04 |

a. Difference in Risk Ratio; b. Difference in Mean
